# Ethnicity- and sex-specific genome wide association study on Parkinson’s disease

**DOI:** 10.1038/s41531-023-00580-3

**Published:** 2023-10-07

**Authors:** Kye Won Park, Ho-Sung Ryu, Eunsoon Shin, YoonGi Park, Sang Ryong Jeon, Seong Yoon Kim, Jae Seung Kim, Seong-Beom Koh, Sun Ju Chung

**Affiliations:** 1 Department of Neurology, Asan Medical Center, University of Ulsan College of Medicine, Seoul, Korea; 2 Pacific Parkinson’s Research Centre, Djavad Mowafaghian Centre for Brain Health, University of British Columbia, Vancouver, Canada; 3 Department of Neurology, Kyungpook National University Hospital, Daegu, South Korea; 4 DNA Link Inc, Seoul, Korea; 5 Department of Neurosurgery, Asan Medical Center, University of Ulsan College of Medicine, Seoul, Korea; 6 Department of Psychiatry, Asan Medical Center, University of Ulsan College of Medicine, Seoul, Korea; 7 Department of Nuclear Medicine, Asan Medical Center, University of Ulsan College of Medicine, Seoul, Korea; 8 Department of Neurology, Korea University Guro Hospital, Seoul, Korea

**Keywords:** Parkinson's disease, Genome-wide association studies

## Abstract

Most previous genome-wide association studies (GWASs) on Parkinson’s disease (PD) focus on the European population. There are several sex-specific clinical differences in PD, but little is known about its genetic background. We aimed to perform an ethnicity-specific and sex-specific GWAS on PD in the Korean population. A total of 1050 PD patients and 5000 controls were included. For primary analysis, we performed a GWAS using a logistic additive model adjusted for age and sex. The same statistical models were applied to sex-specific analyses. Genotyping was performed using a customized microarray chip optimized for the Korean population. Nine single nucleotide polymorphisms (SNPs) including four in the *SNCA* locus and three from the *PARK16* locus were associated with PD in Koreans. The rs34778348 in the *LRRK2* locus showed a strong association, though failed to pass cluster quality control. There were no notable genome-wide significant markers near the *MAPT* or *GBA1* loci. In the female-only analysis, rs34778348 in *LRRK2* and the four other SNPs in the *SNCA* showed a strong association with PD. In the male-only analysis, no SNP surpassed the genome-wide significance threshold under Bonferroni correction; however, the most significant signal was rs708726 in the *PARK16* locus. This ethnicity- and sex-specific GWAS on PD implicate the pan-ethnic effect of *SNCA*, the universal but East-Asian inclined effect of *PARK16*, the East Asian-specific role of *LRRK2* G2385R variants, and the possible disproportionate effect of *SNCA* and *PARK16* between sexes for PD susceptibility. These findings suggest the different genetic contributions to sporadic PD in terms of ethnicity and sex.

## Introduction

Parkinson’s disease (PD) is one of the most common neuro-degenerative disorders, clinically characterized by resting tremor, bradykinesia, rigidity, and postural instability^1^. Its neuropathologic hallmark is a progressive loss of dopaminergic neurons in the substantia nigra caused by pathologic accumulation of α-synuclein, resulting in the formation of Lewy bodies and Lewy neurites^2^.

For the past two decades, genome-wide association studies (GWASs) have shed light on the genetic background of various common diseases including PD^3^. The largest up-to-date meta-analysis of GWASs on PD identified 90 genome-wide significant risk signals across 78 genomic regions which collectively account for 16–36% of the heritable risk of sporadic PD^4^. These PD-related genes were found to be involved in common biological pathways, where several critical cellular routes including mitochondrial dysfunction and lysosomal membrane trafficking pathways lead to pathologic α-synuclein accumulation^2^.

The inveterate problem in the current field of GWASs is the disproportionate focus on European populations^5^. A recent study found that almost 90% of the participants in the National Human Genome Research Institute GWAS Catalog were of European Ancestry^6^. Genetic variants show a high ethnicity-specific heterogeneity in their distribution and functional activity^7^. Thus, the results of previous GWASs targeting European populations cannot readily be generalized to populations with different ethnic or racial backgrounds. Most precedent GWASs on PD have also focused on European descents^4,8^, raising the necessity for the diversity of the target population^9^. Koreans have a distinct genetic makeup in the peninsula owing to their unique geographical and cultural background. Moreover, the prevalence of PD in Korea is expeditiously rising, as it is the world’s most rapidly aging society^10^. Despite this, there is no GWAS data on the Korean PD population.

There are differences in the clinical characteristics of PD according to sex^11^. For example, the prevalence of PD, age at onset, and the susceptibility to progression to dementia differ between males and females. However, little attention has been paid to the genetic differences between male and female patients with PD. A recent sex-specific GWAS on PD conducted in a European population showed no sex-specific differences^12^.

In this context, we aimed to identify the genetic variants associated with PD focusing on a genetic isolate, Koreans, by applying a microarray chip that is optimized for Koreans (Korean Chip) and determine the genomic risk variants for PD in a sex-specific manner.

## Results

### Demographics

A total of 1070 patients with PD and 5000 age- and sex-matched healthy controls were initially recruited in the study. Of them, a sample of 20 patients with PD was excluded due to low sample quality. The mean age at sample collection of the patient group was 64.0 years, ranging from 31 to 89 years. The mean disease duration at study enrollment was 5.3 years. Among them, 554 were female patients. The baseline demographics of the participants including their mini-mental status examination are depicted in Table 1. Power calculation of the sample showed 80% power to detect variants exerting a risk for PD with odds ratio (OR) as low as 1.25 and minor allele frequency (MAF) of 10% (Supplementary Table 1).

**Table 1:** Baseline demographics of the participants in the primary analysis.

Characteristics	Patients (*n* = 1050)	Controls (*n* = 5000)	*P*-value
Female sex	554 (53)	2610 (52)	0.740
Age at sample collection, years	64.0 ± 9.7	64.0 ± 10.0	1.000
Age at onset of Parkinsonism symptoms, years	58.7 ± 10.2	–	
Disease duration at sample collection, years	5.3 ± 4.4	–	
Education duration, years	8.6 ± 6.0	–	
MMSE	26.0 ± 3.5	–	
Disease duration from PD onset to MMSE, years	5.2 ± 4.1	–	

Data are presented as mean ± standard deviation or number of patients (%).

*PD* Parkinson’s disease, *MMSE* mini-mental status examination.

### Primary analysis

**Fig. 1:**
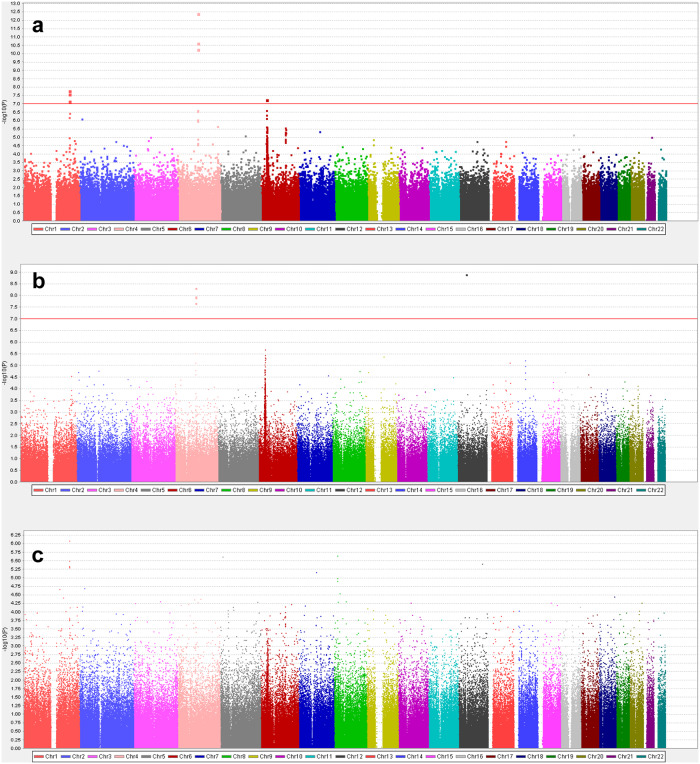
Manhattan plots of the study. Primary analysis (**a**), female-only analysis (**b**), and male-only analysis (**c**). The red lines denote the Bonferroni threshold.

**Fig. 2:**
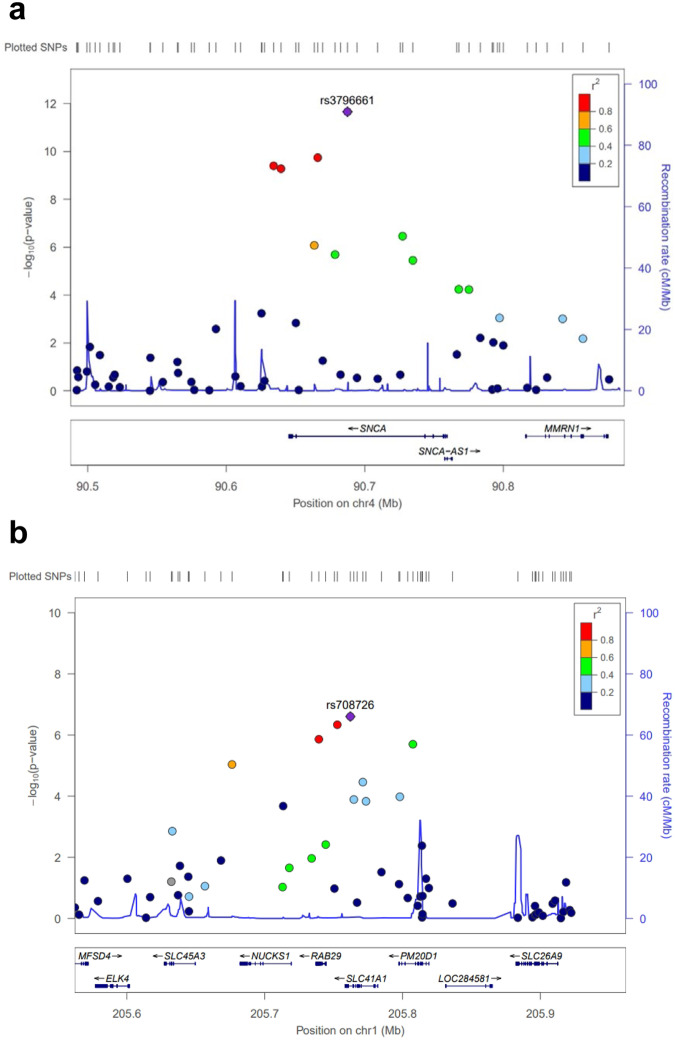
Regional association plots of the primary genome-wide association study. Plots around (**a**) rs3796661 and (**b**) rs708726.

**Fig. 3:**
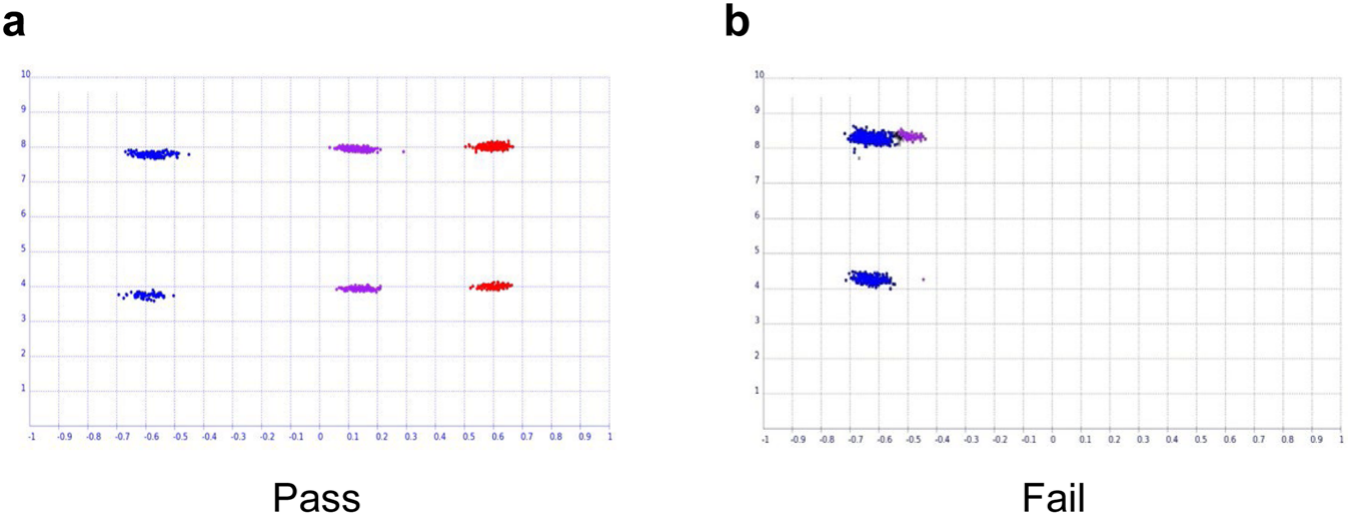
An example of cluster quality control. In general, the three genotypes denoted as blue, purple, and red dots, are clearly clustered (**a**). The markers were excluded if the three genotypes were not clearly separated as in (**b**).

In the primary analysis between 1050 PD patients and 5000 healthy controls, 492,970 single nucleotide polymorphisms (SNPs) passed the marker quality control (QC). The Quantile-Quantile (Q-Q) plot and Manhattan plot of the analysis are shown in Supplementary Fig. 1 and Fig. 1A, respectively. Nine SNPs surpassed the Bonferroni-corrected genome-wide significance, the threshold being 1.01 × 10^–7^ (Table 2). The most strongly associated SNP was rs3796661 (*P* = 3.79 × 10^–13^) in the *SNCA*. Three additional SNPs (rs356203, rs11931074, and rs12640100) in the *SNCA* locus showed significant association with PD. Two SNPs in the *SLC41A1* (rs708726 and rs947211) and *RAB29* (rs708723), which are all located within the *PARK16* locus, were also genome-wide significant. The regional association plots of the *SNCA* locus (index SNP rs3796661) and *PARK16* locus (index SNP rs708725) showed multiple SNPs within the loci in linkage disequilibrium (LD) with the index SNPs (Fig. 2). There were no further SNPs with statistical significance in LD with rs34779348 and rs2451713. Notably, the rs34778348, an exonal missense variant (G2385R) of the *LRRK2* gene, showed a strong association (*P* = 4.77 × 10^–13^). However, the SNP failed to pass the cluster QC that was manually performed after the marker QC steps (Fig. 3). Moreover, when we examined all other markers with *P* < 0.05 in the primary analysis, we could not observe genome-wide significance in the markers near the *MAPT* or *GBA1*, except for the only SNP in the *MAPT* locus (rs374460, *P* = 2.32 × 10^–3^).

**Table 2:** Genomic variants with genome-wide significance of the primary analysis.

Chr	SNP	Gene	Region	Allele (minor/major)	OR (95% CI)	Minor allele frequency (patients/controls)	*P*-value
4	rs3796661	*SNCA*	Intron	C/T	0.69 (0.62–0.76)	0.37/0.46	3.79 × 10^–13^
12	rs34778348	*LRRK2*	Missense, exon	A/G	2.56 (1.99–3.31)	0.05/0.02	4.77 × 10^–13^
4	rs356203	*SNCA*	Intron	T/C	0.71 (0.64–0.79)	0.39/0.46	2.32 × 10^–11^
4	rs11931074	*SNCA, GPRIN3*	Intron, downstream, upstream	G/T	0.72 (0.65–0.79)	0.39/0.47	5.29 × 10^–11^
4	rs12640100	*SNCA, GPRIN3*	Intron, downstream, upstream	G/A	0.72 (0.65–0.79)	0.39/0.47	5.45 × 10^–11^
1	rs708726	*SLC41A1*	Intron	T/G	0.75 (0.68–0.83)	0.43/0.50	1.61 × 10^–8^
1	rs947211	*SLC41A1, RAB29*	Downstream, upstream	A/G	0.75 (0.68–0.83)	0.43/0.50	2.50 × 10^–8^
6	rs2451713	*ZNF322, LOC101929855, GUSBP2*	Upstream, downstream	C/G	1.88 (1.50–2.36)	0.05/0.04	5.39 × 10^–8^
1	rs708723	*RAB29*	UTR-3	C/T	0.76 (0.69–0.84)	0.43/0.49	6.69 × 10^–8^

*Chr* Chromosome, *SNP* single nucleotide polymorphism, *OR* odd’s ratio, *CI* confidence interval, *UTR* untranslated region.

### Sex-specific analysis

In the female-only analysis, 554 female PD patients and 2610 female controls were analyzed. Of the 486,510 SNPs which passed marker QC in the female-only analysis, five SNPs surpassed genome-wide significance threshold under Bonferroni correction (*P* < 1.03 × 10^–7^ (0.05/486,510)) (Table 3, Fig. 1B). The most significant SNP was the rs34778348 in *LRRK2* locus (*P* = 1.25 × 10^–9^). The other four significant SNPs were in the *SNCA*, the most significant being rs3796661 (*P* = 4.89 × 10^–9^). None of the variants in the *PARK16* locus, including those of the *SLC41A1* and *RAB29* genes, had significance under *P* < 1.0 × 10^–5^ in the female-only analysis.

**Table 3:** The most significant genomic variants in the sex-specific GWAS.

Chr	SNP number	Gene	Region	Allele (minor/major)	OR (95% CI)	Minor allele frequency (Patients/Controls)	*P*-value
*Female-only analysis*							
12	rs34778348	*LRRK2*	Missense, exon	A/G	3.53 (2.35–5.29)	0.05/0.02	1.25 × 10^–9^
4	rs3796661	*SNCA*	Intron	C/T	0.63 (0.54–0.74)	0.37/0.46	4.89 × 10^–9^
4	rs12640100	*SNCA, GPRIN3*	Intron, downstream, upstream	G/A	0.64 (0.55–0.75)	0.38/0.47	1.13 × 10^–8^
4	rs356203	*SNCA*	Intron	T/C	0.64 (0.55–0.75)	0.38/0.47	1.25 × 10^–8^
4	rs11931074	*SNCA, GPRIN3*	Intron, downstream, upstream	G/T	0.65 (0.56–0.75)	0.38/0.47	2.11 × 10^–8^
*Male-only analysis*							
1	rs708726	*SLC41A1*	Intron	T/G	0.67 (0.57–0.79)	0.40/0.49	8.23 × 10^–6^
5	rs139422381	*ANKRD33B, LOC101929412*	Downstream, upstream	A/G	4.02 (2.25–7.16)	0.03/0.01	2.46 × 10^–6^
1	rs947211	*SLC41A1, RAB29*	Downstream, upstream	A/G	0.69 (0.59–0.8)	0.41/0.50	3.24 × 10^–6^
12	rs10746109	*WSCD2, LOC728739*	Upstream	A/G	1.46 (1.24–1.71)	0.51/0.44	3.93 × 10^–6^
1	rs708723	*RAB29*	UTR-3	C/T	0.69 (0.59–0.81)	0.40/0.49	4.56 × 10^–6^
1	rs1775145	*SLC41A1, RAB29*	Downstream, upstream	C/A	0.69 (0.59–0.81)	0.42/0.50	4.77 × 10^–6^
1	rs12748961	*NUCKS1, SLC45A3*	Downstream, upstream	T/C	0.69 (0.59–0.81)	0.42/0.50	5.12 × 10^–6^
7	rs1949132	*GNAI1, LOC101927269*	Intron, downstream	C/T	1.78 (1.39–2.29)	0.12/0.09	6.90 × 10^–6^

In the female-only analysis, the genomic variants surpassing the Bonferroni-corrected significance are shown. In the male-only analysis, variants significant with *P* < 10^–6^ but not under Bonferroni correction are shown.

In the male-only analysis, 496 male PD patients and 2390 male controls were included. A total of 488,631 SNPs passed the marker QC. None of the SNPs surpassed the genome-wide significance threshold under Bonferroni correction (*P* < 1.02 × 10^–7^ (0.05/488,631); Table 3, Fig. 1C). However, when the top signals’ *P*-value under 1.0 × 10^–6^ were reviewed, the most significant signal was the rs708726 in the *SLC41A1* (*P* = 8.23 × 10^–6^), with four others in the *PARK16* locus with *P* < 1.0 × 10^–6^. Meanwhile, SNPs within the *SNCA* locus did not show associations with *P*-value < 10^–4^ except for rs3796661 (*P* = 5.25 × 10^–5^), indicating its small effect on male patients compared to female patients. The demographics, power calculation, Q-Q plots, and regional association plots of these sex-specific analyses can be found in the supplementary materials (Supplementary Tables 2, 3, Supplementary Figs. 2, 3). The least OR to satisfy the statistical power of 80% at MAF of 10% in the female and male subgroups was 1.34 and 1.36, respectively.

## Discussion

In this ethnicity-specific GWAS on PD, variants in the *SNCA* and *PARK16* loci showed the strongest association with PD in the Korean population. We further found that the *LRRK2* G2385R variant was associated with Korean PD, although the variant did not pass cluster QC. Variants in *MAPT* or *GBA1*, the two genes commonly associated with PD in GWASs from Western countries^13^, were not replicated in our GWAS. There were disproportionate effects of *SNCA* and *PARK16* variants on Korean PD according to sex. Although we did not identify any novel loci specific to Korean ethnicity for PD susceptibility, our results suggest that there is a gradient in genetic contribution according to ethnicity and sex to the risk of PD.

Our dominant SNPs being in the *SNCA* locus demonstrates the universal strong effect of the *SNCA* variants on the risk of PD across ethnicities. Abnormal accumulation of the α-synuclein protein, which normally regulates synaptic vesicle trafficking and the subsequent neurotransmitter release in neurons, is the pathological hallmark of PD^14,15^. The *SNCA* gene, which encodes the α-synuclein protein, was identified as the risk loci from the first large-scale GWAS on PD^16^. The following GWASs on PD targeting various populations and subsequent meta-analyses consistently reported strong effects of the loci variants on the risk of PD, regardless of the target population^4,17,18^. In contrast, the effect of the second leading loci in our study, *PARK16*, including the SNP rs708726, has been particularly highlighted in GWASs on PD targeting East Asians. The locus spans across five genes, including *SLC45A3, NUCKS1, RAB29/RAB7L1, SLC41A1*, and *PM20D1*^19^. Among these regions, *RAB29* is the master regulator of the LRRK2 protein, controlling its activation, localization, and phosphorylation^20^. The locus was designated with the name *PARK16* after the discovery of its association with PD in a Japanese GWAS^21^. The largest GWAS on PD in the East Asian population thus far by Foo and colleagues, which was performed on more than 30,000 participants across six populations of East Asia, found that *PARK16* is a dominant locus in East Asian PD, along with the *SNCA* and *LRRK2* loci^18^. In line with these previous studies, our study suggests that the effect of PARK16 variants on PD susceptibility is pan-ethnic but particularly stands out in East Asians.

In this study, the strong association of rs34778348 with PD in *LRRK2* was another notable finding. SNP rs34637584, known as the *LRRK2* G2019S variant, is a well-known variant that is strongly associated with PD risk in the Caucasian and Jewish populations^22^. In contrast to this, rs34778348, which is the *LRRK2* G2385R variant, is mainly found in Asian populations^23^. This variant was found to be a genetic risk factor for sporadic PD in Chinese, Japanese, and Korean populations^23–25^. A previous study on *LRRK2* G2395R in Korean PD included only a small number of participants^24^, and our study provides a replication of their findings in a larger sample size. However, a careful interpretation is warranted because this variant failed to pass the cluster QC and no other markers in LD with the variant were shown to be significant in our analysis. The kinase overactivity and downregulation of the *LRRK2* function with kinase inhibitors caused by the *LRRK2* G2019S variant has been suggested as a potential therapeutic target of PD^26,27^. It has been proposed that *LRRK2* G2385R results in partial loss-of-function of the kinase activity in vitro^28^, in contrast to its G2019S counterpart. The discrepancy in the *LRRK2* variants and subsequent protein dysfunction between Caucasian and Asian populations is of great importance, warranting different therapeutic approaches according to ethnicity.

We could not observe evidence for associations with the *GBA1* or *MAPT* loci, which are the two important genes highlighted in both sporadic and familial PD in Western countries^29^. One possible explanation is the high homogeneity of *MAPT* in the East Asian population. In East Asia, *MAPT* is genetically homogenous with only the H1 haplotype in the population, whereas the European population has both H1 and H2 haplotypes^30^. However, multiple variants exist even in the H1 haplotype, reflecting the greater diversity of *MAPT* than explained by the H1 and H2 clades alone^31^. Thus, the lack of association with *MAPT* or *GBA1* in this Korean-specific analysis may suggest the difference in the susceptibility to PD by the variants within these genes. The associations of the two loci were also not replicated by other Asian GWASs on PD, including those in Japanese^23^ Han-Chinese^25^ and in pan-East Asian GWASs on PD^18,32^, supporting our findings.

Investigation regarding the genes associated with PD in a sex-specific manner has been limited. In our analysis, rs34778348 of the *LRRK2* locus and four SNPs of the *SNCA* locus showed genome-wide significance in females, but the significance was not replicated in males. The most significant SNPs in males were those in the *PARK16* locus whereas they did not surpass the significance threshold under the Bonferroni correction. However, a recent investigation on autosomal genetic and sex-specific differences in PD found no significant genetic differences between male or female PD patients^12^. PD is more prevalent in men worldwide but is more prevalent in women in Asian populations, including the Korean population^11^. The discrepancy between the European and Korean sex-specific GWASs on PD may implicate such ethnicity-specific differences in the sex ratio of PD.

There are some limitations in our study. First, the analysis was conducted without principal component adjustment under the assumption of genetic homogeneity of the Korean population. Ample evidence supports that many of the Far East Asian population groups, especially the Korean, have their own distinct genetic cluster without population admixture^33,34^. Although we presented the genetic homogeneity of our dataset in Supplementary Fig. 4, without adjusting the principal components in the analyses, potential stratification at the sub-populational level cannot be ruled out. Second, the number of total subjects was relatively small for a GWAS hence underpowering the results, especially when stratified by sex. Nevertheless, it is an inevitable limitation for a genetic study targeting a minor genomic cluster. Third, our study lacks functional validation of the discovered variants and a separate replication analysis. On the other hand, there have been several well-designed meta-analyses of GWASs on PD with various methods of functional validations^35^. Our study did not reveal any novel marker specific to Korean PD. Thus, in a way, our work itself could be interpreted as a Korean validation of the worldwide level meta-analyses. To identify the ethnic-sex-genetic interaction suggested in this study, further functional validation specific to ethnicity and sex should be encouraged.

This ethnicity- and sex-specific GWAS on PD in the Korean population suggests the pan-ethnic effect of *SNCA*, the standing out significance of *PARK16* in East Asians, the East Asian-specific role of the *LRRK2* G2385R variants, and the possible disproportionate effect of *SNCA* and *PARK16* between sexes for PD susceptibility. These findings implicate the gradient in genetic contribution to PD susceptibility across ethnicities and sex.

## Methods

### Participants

We recruited patients with PD in Asan Medical Center, Seoul, South Korea from January 2011 to April 2016. A total of 1070 ethnically Korean patients who were diagnosed with sporadic PD by movement disorder specialists according to the United Kingdom Parkinson’s Disease Brain Bank Criteria were enrolled^36^. Baseline demographics including age at sample collection, age at the onset of PD, sex, and family history of Parkinsonism were collected. We defined the age at onset as the time when one of the motor cardinal symptoms (resting tremor, rigidity, bradykinesia, stooped posture, or postural instability) was noted by the patient or caregiver. Exclusion criteria were as follows: not being ethnically Korean, genetically confirmed hereditary Parkinsonism, and signs of atypical Parkinsonism (cerebellar signs, Parkinsonism not-responsive to levodopa, supranuclear gaze palsy, early severe autonomic dysfunction, early severe dementia with disturbances of memory, language, and praxis, and otherwise-unexplained pyramidal signs). For controls, we obtained the samples of 5000 age- and sex-matched healthy controls from the Korea Biobank Project. Informed consent was obtained from every participant as per the locally approved protocols. The study was approved by the Institutional Review Board of Asan Medical Center.

### Genotyping and quality control

All patients underwent peripheral blood sampling for DNA extraction, and 200 ng of genomic DNA was genotyped for each patient. All samples were genotyped on a Korean Chip (K-CHIP) obtained from the K-CHIP Consortium, Center for Genome Science, Korea National Institute of Health^37^. The K-CHIP is an SNP microarray chip developed to standardize the genotypic platform optimal for Koreans. All samples were assayed on Affymetrix Axiom® 2.0 Reagent Kit (Affymetrix, Santa Clara, CA, USA). Manual target preparation for the assay was processed according to the manufacturer’s protocol. Low-quality samples and low-quality SNPs were excluded through the following QC steps. Samples with call rates lower than 97%, sex discrepancy, excessive heterozygosity, or cryptic relatedness were excluded. SNPs with minor allele frequency <1% in patients or controls, markers with a low call rate <95% in patients or controls, and SNPs with significant deviation from the Hardy–Weinberg equilibrium permutation test (*P* < 10^–4^) were excluded. Lastly, all markers with *P* < 10^–4^ were visually inspected by one of the investigators for cluster QC (Fig. 3). For each marker, the genotypes (AA, Aa, or aa) were colored red, purple, and blue, respectively. If the genotypes of a marker were not clearly clustered into the three colors, we considered it as genotyping error and the marker was excluded. All QC steps were performed using PLINK software version 1.90 (Free Software Foundation Inc., Boston, MA, USA)^38,39^.

### Statistical analysis

We performed primary analysis between PD patients and healthy controls by multiple logistic additive models with age and sex as the covariates. Age at onset of Parkinsonism for the PD patients and age at sample collection for the healthy controls were adjusted as the age covariate. Secondary analyses, the sex-specific GWASs, were performed in the male and female population separately using the same model and covariates. Principal component analysis (PCA) was performed to verify the populational homogeneity of our dataset (Supplementary Fig. 4). PLINK software version 1.90 (https://www.cog-genomics.org/plink2/) was used for the association analysis^38,39^. Q–Q plots and Manhattan plots were contrived using the R software (version 3.5.2, R Core Team (2018). R: A language and environment for statistical computing. R Foundation for Statistical Computing, Vienna, Austria. URL https://www.R-project.org/). Regional association plots were generated using the LocusZoom software (version 0.4.8, http://locuszoom.sph.umich.edu/)^40^. Power calculations were performed using Quanto software (version 1.2.4.)^41^. Regional association plots were generated and visually inspected in all SNPs with *P* < 10^–4^. Bonferroni corrections were applied to correct multiple tests.

### Reporting summary

Further information on research design is available in the Nature Research Reporting Summary linked to this article.

### Supplementary information

Reporting summary

supplementary materials

## Data Availability

The summary statistics of this GWAS are openly available in GWAS Catalog (https://www.ebi.ac.uk/gwas/downloads/summary-statistics, study accession: GCST90278092).

## Code availability

The related codes and scripts for the study will be made available upon reasonable request to the corresponding author.
