# Supplementary material for: Ethnicity- and sex-specific genome wide association study on Parkinson’s disease
Source: NPJ Parkinsons Dis. 2023 Oct 7;9:141. doi: 10.1038/s41531-023-00580-3 (PMC10560250; doi:10.1038/s41531-023-00580-3)
Supplement: Supplementary file 1 — Reporting summary [file 41531_2023_580_MOESM1_ESM.pdf]

nature portfolio

Corresponding author(s): Sun Ju Chung

Last updated by author(s): Sep 11, 2023

Reporting Summary

Nature Portfolio wishes to improve the reproducibility of the work that we publish. This form provides structure for consistency and transparency in reporting. For further information on Nature Portfolio policies, see our Editorial Policies and the Editorial Policy Checklist.

Statistics

For all statistical analyses, confirm that the following items are present in the figure legend, table legend, main text, or Methods section.

n/a	Confirmed	
☐	☒	The exact sample size (*n*) for each experimental group/condition, given as a discrete number and unit of measurement
☐	☒	A statement on whether measurements were taken from distinct samples or whether the same sample was measured repeatedly
☐	☒	The statistical test(s) used AND whether they are one- or two-sided<br>*Only common tests should be described solely by name; describe more complex techniques in the Methods section.*
☐	☒	A description of all covariates tested
☐	☒	A description of any assumptions or corrections, such as tests of normality and adjustment for multiple comparisons
☐	☒	A full description of the statistical parameters including central tendency (e.g. means) or other basic estimates (e.g. regression coefficient) AND variation (e.g. standard deviation) or associated estimates of uncertainty (e.g. confidence intervals)
☐	☒	For null hypothesis testing, the test statistic (e.g. *F*, *t*, *r*) with confidence intervals, effect sizes, degrees of freedom and *P* value noted<br>*Give P values as exact values whenever suitable.*
☒	☐	For Bayesian analysis, information on the choice of priors and Markov chain Monte Carlo settings
☒	☐	For hierarchical and complex designs, identification of the appropriate level for tests and full reporting of outcomes
☒	☐	Estimates of effect sizes (e.g. Cohen's *d*, Pearson's *r*), indicating how they were calculated

Our web collection on statistics for biologists contains articles on many of the points above.

Software and code

Policy information about availability of computer code

Data collection	n/a
Data analysis	All samples were assayed on Affymetrix Axiom® 2.0 Reagent Kit (Affymetrix, Santa Clara, CA, USA).<br>Sample quality control, marker quality control, and the association analyses were were performed using PLINK software version 1.90 (Free Software Foundation Inc., Boston, MA, USA).<br>Principal component analysis, Q-Q plots, and Manhattan plots were performed and plotted using R software version 3.5.2 (R Core Team, Vienna, Austria).<br>Regional association plots were generated using the LocusZoom software version 0.4.8 (University of Michigan, Department of Biostatistics, Center for Statistical Genetics).<br>Power calculations were performed using Quanto software version 1.2.4 (Keck School of Medicine of University of Southern California, Department of Population and Public Health Sciences).

For manuscripts utilizing custom algorithms or software that are central to the research but not yet described in published literature, software must be made available to editors and reviewers. We strongly encourage code deposition in a community repository (e.g. GitHub). See the Nature Portfolio guidelines for submitting code & software for further information.

Data

Policy information about availability of data

All manuscripts must include a data availability statement. This statement should provide the following information, where applicable:

- Accession codes, unique identifiers, or web links for publicly available datasets
- A description of any restrictions on data availability
- For clinical datasets or third party data, please ensure that the statement adheres to our policy

The summary statistics of this GWAS are openly available in GWAS Catalog (https://www.ebi.ac.uk/gwas/downloads/summary-statistics, study accession: GCST90278092).

Research involving human participants, their data, or biological material

Policy information about studies with human participants or human data. See also policy information about sex, gender (identity/presentation), and sexual orientation and race, ethnicity and racism.

Reporting on sex and gender	The term sex was used in this study to reflect biological attribute at birth as patient report or documented in the medical records.
Reporting on race, ethnicity, or other socially relevant groupings	Our study did not use socially constructed or socially relevant categorization variables.
Population characteristics	The population in the analyses included 1,050 patients with PD (mean age 64 years, range 31-89 years) and 5,000 age- and sex-matched healthy controls. The mean disease duration of patient group at study enrollment was 5.3 years.
Recruitment	Patients were recruited in Asan Medical Center, Seoul, South Korea from January 2011 to April 2016. Control data were obtained from the Korea Biobank Project.
Ethics oversight	Informed consent was obtained from every participant as per the locally approved protocols. The study was approved by the Institutional Review Board of Asan Medical Center.

Note that full information on the approval of the study protocol must also be provided in the manuscript.

Field-specific reporting

Please select the one below that is the best fit for your research. If you are not sure, read the appropriate sections before making your selection.

☒ Life sciences ☐ Behavioural & social sciences ☐ Ecological, evolutionary & environmental sciences

For a reference copy of the document with all sections, see nature.com/documents/nr-reporting-summary-flat.pdf

Life sciences study design

All studies must disclose on these points even when the disclosure is negative.

Sample size	A total of 1,050 patients with PD and 5,000 healthy controls were included in the genome-wide association study. . Power calculation of the sample showed 80% power to detect variants exerting a risk for PD with odds ratio as low as 1.25 and minor allele frequency of 10%.
Data exclusions	Samples with call rates lower than 97%, sex discrepancy, excessive heterozygosity, or cryptic relatedness were excluded. SNPs with minor allele frequency less than 1% in patients or controls, markers with low call rate less than 95% in patients or controls, and SNPs with significant deviation from Hardy-Weinberg equilibrium permutation test (P < 10E-4) were excluded. Markers without clear visual clustering into three colors in cluster analyses were excluded.
Replication	The study did not include a replication analysis.
Randomization	The study is a case-control study. Covariates including age and sex were adjusted. Principal component analysis was performed to verify the population homogeneity of the dataset.
Blinding	The investigators were blinded to the genotypes when carrying out any data analyses.

Reporting for specific materials, systems and methods

We require information from authors about some types of materials, experimental systems and methods used in many studies. Here, indicate whether each material, system or method listed is relevant to your study. If you are not sure if a list item applies to your research, read the appropriate section before selecting a response.

Materials & experimental systems

n/a	Involved in the study
☒	☐ Antibodies
☒	☐ Eukaryotic cell lines
☒	☐ Palaeontology and archaeology
☒	☐ Animals and other organisms
☒	☐ Clinical data
☒	☐ Dual use research of concern
☒	☐ Plants

Methods

n/a	Involved in the study
☒	☐ ChIP-seq
☒	☐ Flow cytometry
☒	☐ MRI-based neuroimaging
