# Supplementary material for: Ethnicity- and sex-specific genome wide association study on Parkinson’s disease
Source: NPJ Parkinsons Dis. 2023 Oct 7;9:141. doi: 10.1038/s41531-023-00580-3 (PMC10560250; doi:10.1038/s41531-023-00580-3)
Supplement: Supplementary file 2 — supplementary materials [file 41531_2023_580_MOESM2_ESM.pdf]

Supplementary Table 1. Power calculation of the primary analysis

Minor Allele Frequency	Least OR to satisfy statistical power of 80%
0.05	1.34
0.10	1.25
0.15	1.20
0.20	1.18
0.25	1.18
0.30	1.17
0.35	1.17
0.40	1.16
0.45	1.16
0.50	1.16

Abbreviation: OR, odd's ratio.

Supplementary Table 2. Demographics of the sex-specific analyses

Characteristics	Patients	Controls	P-value
Female sex	*(n = 554)*	*(n = 2,610)*	
Age at sample collection, years	64.0 ± 9.2	64.0 ± 9.0	1.000
Age at onset of PD, years	58.6 ± 9.8	-	-
Disease duration at sample collection, years	5.4 ± 4.8	-	-
Education duration, years	7.2 ± 5.5	-	-
MMSE	25.5 ± 3.7	-	-
Disease duration from PD onset to MMSE, years	5.4 ± 4.4	-	-
Male sex	*(n = 496)*	*(n = 2,390)*	
Age at sample collection, years	64.0 ± 10.2	64.0 ± 9.0	1.000
Age at onset of PD, years	58.8 ± 10.6	-	-
Disease duration at sample collection, years	5.1 ± 4.0	-	-
Education duration, years	7.2 ± 5.5	-	-
MMSE	26.5 ± 3.0	-	-
Disease duration from PD onset to MMSE, years	5.1 ± 3.9	-	-

Supplementary Table 3. Power calculation of the sex-specific analysis

Minor allele frequency	Least OR to satisfy statistical power of 80%	
	Female sex	Male sex
0.05	1.47	1.50
0.10	1.34	1.36
0.15	1.28	1.30
0.20	1.26	1.27
0.25	1.24	1.26
0.30	1.23	1.24
0.35	1.22	1.24
0.40	1.21	1.23
0.45	1.21	1.23
0.50	1.21	1.23

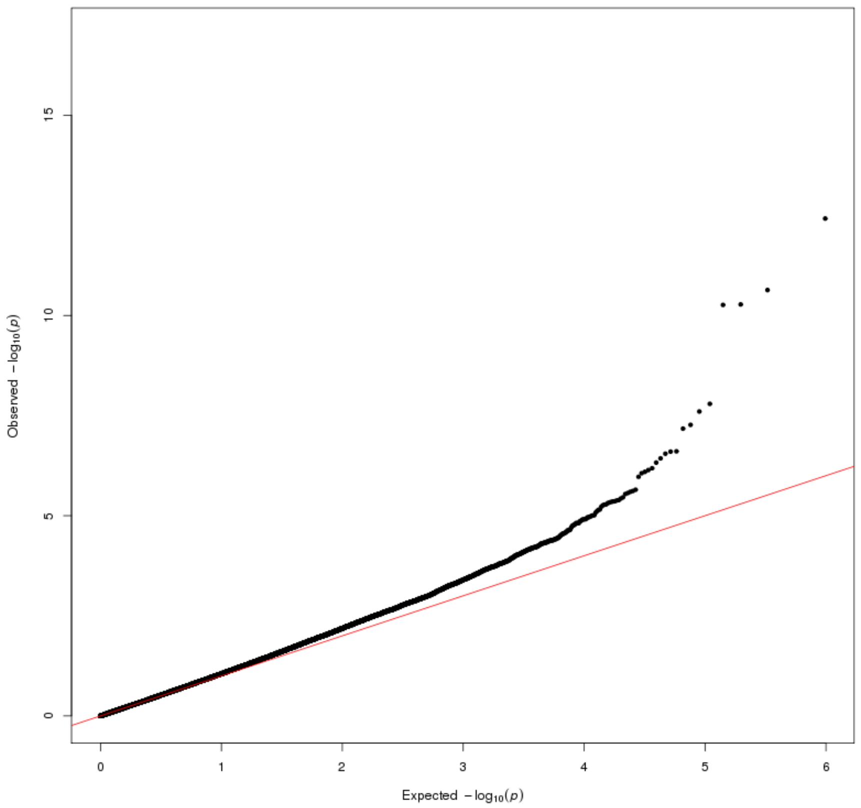

Supplementary Fig. 1. Quantile-quantile plot of the primary analysis.

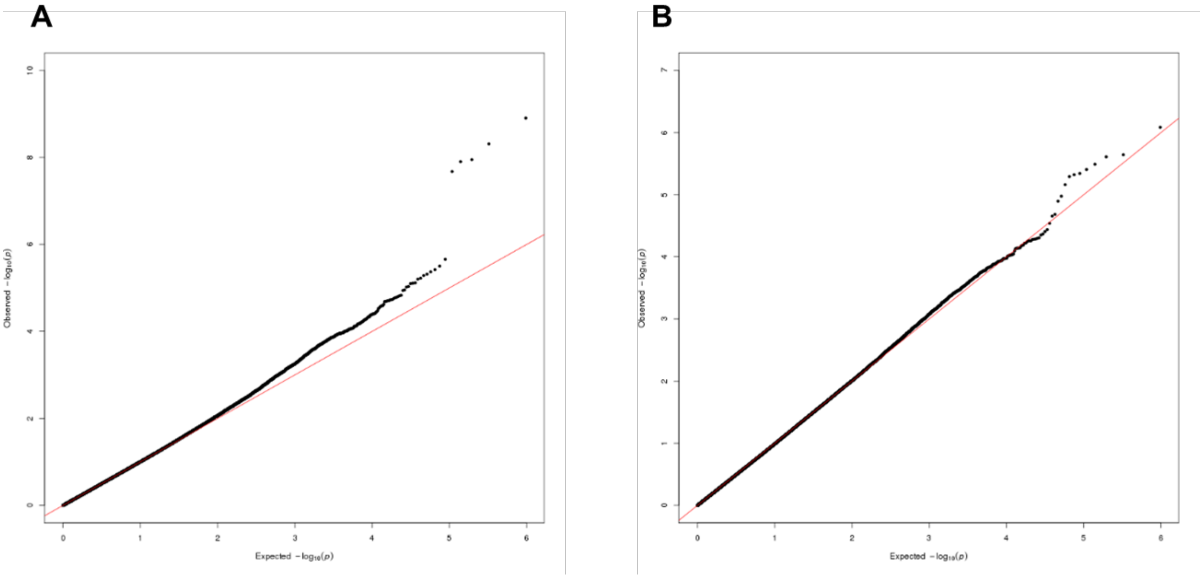

Supplementary Fig. 2. Quantile-quantile plot of the sex-specific analyses. (A) Female sex, and (B) male sex.

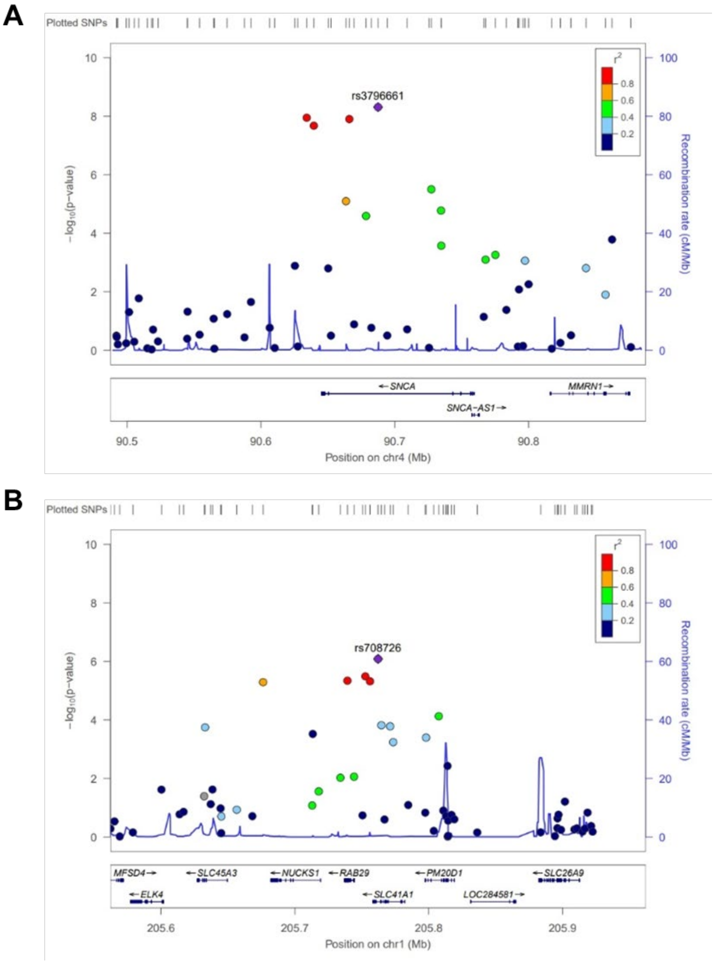

Supplementary Fig. 3. Regional association plot around (A) rs379661 in the female-only analysis, and (B) rs708726 in the male-only analysis.

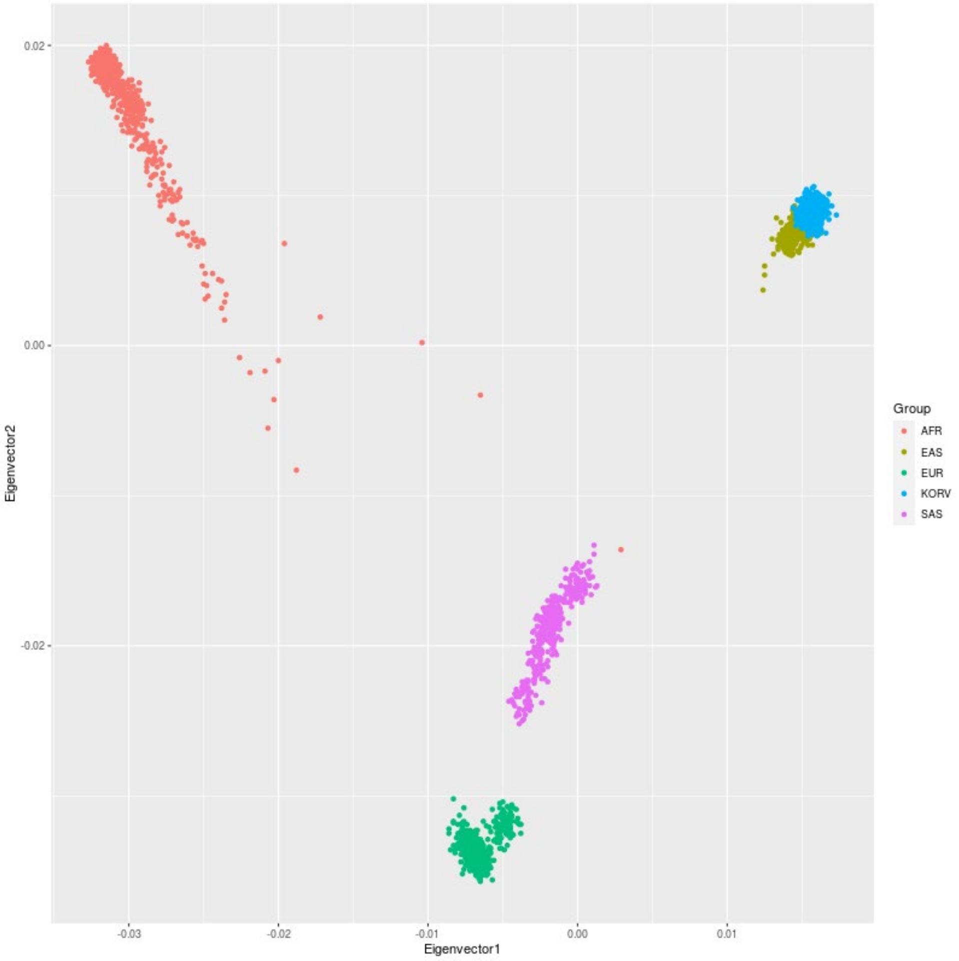

Supplementary Fig. 4. Principal Component Analysis (PCA) plot showing the population structure based on a merged dataset consisting of our Korean dataset (KORV) and other superpopulations included in the 1000Genome dataset (AFR, African; EAS, East Asians; EUR, European; SAS, South Asian). Ad-Mixed American superpopulation of the 1000Genome dataset is not presented in the plot due to its high dispersion.
